# Longitudinal Variations of *M. tuberculosis*-Induced IFN-γ Responses in HIV-Negative Pregnant Women Exposed to Tuberculosis

**DOI:** 10.3389/fimmu.2021.805157

**Published:** 2021-12-22

**Authors:** Paulo Ranaivomanana, Rila Ratovoson, Crisca Razafimahatratra, Arimanitra Razafimahefa, Jonathan Hoffmann, Perlinot Herindrainy, Julio Rakotonirina, Niaina Rakotosamimanana

**Affiliations:** ^1^ Mycobacteria Unit, Institut Pasteur de Madagascar, Antananarivo, Madagascar; ^2^ Epidemiology Unit, Institut Pasteur de Madagascar, Antananarivo, Madagascar; ^3^ Medical and Scientific Department, Fondation Mérieux, Lyon, France; ^4^ Centre Hospitalier Universitaire de Soins et Santé Publique Analakely (CHUSSPA), Antananarivo, Madagascar

**Keywords:** tuberculosis, interferon-gamma release assays (IGRA), pregnancy, human whole blood, QuantiFERON-TB Gold Plus^®^

## Abstract

**Introduction:**

Pregnancy triggers an alteration of the immune functions and increases the risk of developing the active tuberculosis (TB) symptoms in exposed women. The effect of pregnancy on the *Mycobacterium tuberculosis-*specific immune responses used for most of the TB immunodiagnostic assays is not well documented. Here we investigated the changes in the *M. tuberculosis*-specific IFN-γ production in age-matched pregnant and non-pregnant women according to their TB exposition status.

**Methods:**

We conducted a prospective cohort study on HIV-seronegative pregnant and non-pregnant women with compatible pulmonary TB symptoms addressed to TB healthcare facilities in Antananarivo, Madagascar. Active pulmonary TB was bacteriologically assessed with culture from sputum samples. Clinical data and blood samples were collected at inclusion and after 6 months of follow-up for each individual included. Whole blood samples were stimulated with QuantiFERON TB-Gold Plus (QFT-P) assay antigens. Plasma IFN-γ concentrations were then assessed by ELISA.

**Results:**

A total of 284 women were investigated for the study including 209 pregnant women without confirmed TB (pNTB), 24 pregnant women with bacteriologically confirmed active TB (pATB), 16 non-pregnant women with active TB (ATB), and 35 non-pregnant healthy donors (HC).

At inclusion, IFN-γ responses are lower in the pregnant women compared to their age-matched non-pregnant counterparts and independently of their TB status. Among the pregnant women, higher concentrations of *M. tuberculosis*-specific IFN-γ were observed in those exposed to TB, but with a lower magnitude in the active TB compared to the latently infected pregnant women (p < 0.05 with TB1 and p < 0.01 with TB2). After 6 months of follow-up, the *M. tuberculosis*-specific IFN-γ responses return to their baseline concentrations except for the pregnant women treated for TB for which none of the QFT-P positive reversed to negative (0%, 0/10) at the end of their TB treatment.

**Conclusion:**

These results support the concept of specific immune priorities characterized by a concomitant reduction in inflammatory immunity during pregnancy and corroborate the important role of activating the *M. tuberculosis-*specific immune responses to control the infection when the pregnant women are exposed to the pathogen.

## Introduction

Tuberculosis (TB) remains a public health threat worldwide with an estimated 10 million notified cases and 1.4 million deaths every year (1). TB is an important cause of maternal mortality and morbidity globally, and the number of TB cases in women of reproductive age (15–49 years) is increasing (1). Particularly, TB in pregnancy was found to be associated with increased risks of pregnancy complications, prematurity, neonatal morbidity, low birth weight, and perinatal deaths (2, 3).

The physiological changes during pregnancy result in distinct immune responses that lead to an immunological tolerance toward the fetus characterized by downregulations of both the cell-mediated immunity and the production of Th1 cytokines and an upregulation of both the humoral immunity and the Th2 cytokine productions (4, 5). While this maternal immune tolerance protects the fetus from rejection and promote a healthy pregnancy outcome, it may increase the vulnerability against pathogens like TB as the cell-mediated immunity and the Th1 response play key roles in containing the *M. tuberculosis* infection (6, 7).

Interferon-gamma release assays (IGRAs) are immunoassays based on the measure of the production of IFN-γ produced after T cell *ex vivo* stimulation by *M. tuberculosis*-specific antigens also used to assess for a previous exposure or infection to *M. tuberculosis*. Recent studies on the IFN-γ response changes in *M. tuberculosis* latently infected pregnant women (pLTBI+) using IGRAs suggested a slight increase of the Th1 pro-inflammatory response to a concentration sufficient to contain the latent infection in these pregnant women (7–9 and 10). However, few data are available on this IFN-γ immune response status in pregnant women with active TB disease.

In the present study, we investigated the *M. tuberculosis*-specific immune response in pregnant women with active TB disease. Moreover, we assess the IFN-γ response changes postpartum and/or following a successful TB treatment and compare them to those from non-pregnant women with or without active TB disease.

## Methods

### Study Design and Setting

Study participants were included from two studies: a cross-sectional prospective cohort study for non-pregnant women and one cohort survey for pregnant women conducted between April 2018 to August 2019 in primary healthcare facilities in the capital city of Antananarivo, Madagascar [as previously described in 11 and Chedid et al., 2012 (12)]. Pregnant women with TB-compatible symptoms defined as one or a combination of cough more than 2 weeks, hemoptysis, dyspnea, chest pain, weight loss, night sweats, loss of appetite, fever, and deterioration of general condition were invited to participate. Non-pregnant women with presumptive TB symptoms were also recruited if diagnosed with bacteriologically confirmed pulmonary TB (ATB) (see *Mycobacteriology* section for further detail).

To investigate the baseline responses for *M. tuberculosis* non-exposed individuals, age-matched community control non-pregnant women without symptoms or signs of TB and no known recent or sustained contact with TB cases (HC) were recruited at the post-exposure rabies center of Institute Pasteur de Madagascar (Antananarivo, Madagascar).

Patients with HIV (human immunodeficiency virus) or diabetes mellitus and participants under 15 years were excluded from the study. In downstream analyses, patients under immunocompromising treatment (corticosteroids, calcineurin inhibitors, biologics, or other chemotherapeutic agents) were excluded from the analyses.

### Data Collection and Laboratory Procedures

Sociodemographic characteristics, TB history, pregnancy-related factors (i.e., gestational age, parity), Bacillus Calmette–Guérin (BCG) vaccinal status, comorbidities (HIV, hepatitis), and data on TB-presumptive clinical symptoms were collected using standardized questionnaires by trained nurses.

#### Mycobacteriology

Confirmed TB cases were defined as smear microscopy positive for AFB (Ziehl–Neelsen and/or Auramine staining) and Lowenstein–Jensen solid medium culture positive for *M. tuberculosis* from sputum samples.

#### Interferon-Gamma Assay

A 5-ml venous blood sample was drawn in a blood-collecting tube containing lithium heparin for each participant. The blood samples were transported to the laboratory within 4 h after blood sampling at the controlled temperature (22 ± 5°C), and a 1-ml sample was dispensed into each assay tube, followed by mixing by inversion, and incubated at 37°C for 16–20 h within 16 h after collection. After incubation, the tubes were centrifuged, and aliquots of the supernatants were stored at -20°C.

IFN-γ secretion was quantified using the QFT-P ELISA Kit (Qiagen) according to the manufacturer’s instructions (13). Briefly, plasma samples were thawed at room temperature, and 50 µl of plasma was tested. Optical density results were compared to log-normalized values from freshly reconstituted IFN-γ kit standards. To account for potential immunomodulation phenomena, baseline IFN-γ concentration values (NIL tubes) were subtracted from antigen-stimulated IFN-γ values (MIT, TB1, TB2). The NIL tube contained no antigens and was used as a negative control. The TB1 and TB2 QFT-P tubes are coated with commercial *M. tuberculosis*-specific antigenic peptide pools. TB1 tubes contain two mycobacterial peptides, ESAT-6 (>15 aa) and CFP-10, which elicit specific immune responses from CD4+ T lymphocytes (13). TB2 tubes contain an additional commercial peptide pool designed to induce CD8+ T lymphocyte stimulation. MIT tubes are coated with commercial phytohemagglutinin-like bacterial antigens and were used as a positive control (13).

According to the kit’s sensitivity range, the maximum for the IFN-γ concentration value was set at 10 IU/ml and negative values were rescaled to 0; the cutoff value for a positive test was 0.35 IU/ml.

### Follow-Up Visits

Each participant except the HC group was followed up at inclusion (D0) and at 6 months (M6) where QFT-P tests were performed. Confirmed TB patients were put on Directly Observed Treatment (DOT) and received treatment according to standard WHO protocols (14). Briefly, for the TB-confirmed participants, the treatment consisted of 2 months of daily rifampicin, isoniazid, pyrazinamide, and ethambutol, followed by 4 months of daily rifampicin and isoniazid. The successful TB treatment course is assessed based on the resolution of clinical symptoms and sputum conversion to negativity (microscopy and culture) according to the Malagasy national and WHO guidelines. For the pregnant women, all maternal and fetal outcomes were collected including delivery.

### Statistical Analysis

All data generated from the study were recorded in a REDCap^®^ software database. GraphPad Prism version 8.0 was used for statistical analyses. The χ2 and Fisher exact tests were performed to compare categorical variables between groups, as appropriate. The Mann–Whitney test was used to analyze the magnitude differences of IFN-γ responses between groups. Pairwise comparisons of *M. tuberculosis*-triggered IFN-γ concentrations were performed between DO and M6 separately for the two antigens (TB1 and TB2) using the Wilcoxon matched-pair signed-rank test. A p-value <0.05 was considered statistically significant.

### Ethical Considerations

All information about the study was explained in local language; written informed consent and completed questionnaires were obtained from all included participants. The methods were carried out in accordance with the approved guidelines. The 2 studies were approved by the Ethical Committee for Biomedical Research in Madagascar (reference number: 099 MSANP/CERBM 2018 and 103MSANP/CERBM 2017).

## Results

### Study Participant Characteristics

A total of 284 women were included in the study including 209 pregnant women without bacteriologically confirmed TB (pNTB), 24 pregnant women with active TB (pATB), and 16 non-pregnant women with active TB (ATB) (**Table 1**). Moreover, thirty-five (35) non-pregnant healthy women donors (HD) were also recruited as controls. There was no significant difference in the age between pregnant and non-pregnant groups. Among pregnant women (pNTB and pATB), 91% of the studied population had been vaccinated with BCG at childhood without significant difference between pregnant and non-pregnant women (**Table 1**).

**Table 1:** Baseline characteristics of the study participants.

Characteristics	pNTB n = 202	pATB n = 24	ATB n = 16	p value
**Age (mean SD)**	26.2 (5.4)	25.7 (5.3)	28.8 (12.1)	>0.05
Gravidity				
1	70 (34.7)	11 (45.8)	–	0.55
2	39 (19.3)	4 (16.7)	–	
≥3	93 (46.0)	9 (37.5)	–	
**Marital status**				
Married	77 (38.1)	8 (34.8)	-	0.86
Free union	120 (59.4)	15 (65.2)	-	
Single	4 (2.0)	0 (0.0)	-	
Divorced/widowed	1 (0.5)	0 (0.0)	-	
**Educational level**				
No	9 (4.5)	0 (0.0)	0 (0.0)	0.80
Primary	56 (27.7)	5 (21.7)	6 (37.5)	
Secondary	112 (55.4)	15 (65.2)	8 (50.0)	
University	25 (12.4)	3 (13.0)	2 (12.5)	
**Occupational status**			n/a	
Unemployed	79 (39.1)	11 (47.8)	6 (37.5)	<0.0001
Manual work	120 (59.4)	11 (47.8)	7 (43.8)	
Work in an office	0 (0.0)	1 (4.3)	0(0.0)	
Student	3 (1.5)	0(0.0)	3 (18.8)	
**TB contact history**				
Yes	37 (18.3)	12 (50.0)	9 (56.1)	<0.0001
No	163 (80.7)	11 (45.8)	2 (12.5)	
Unknown	2 (1.0)	1 (4.2)	5 (31.3)	
**TB treatment history**				
Yes	12 (5.9)	3 (12.5)	3 (18.7)	0.10
No	190 (94.1)	21 (87.5)	13 (81.3)	
**BCG vaccination**				
Yes	185 (91.5)	21 (91.4)	14 (87.4)	0.97
No	7 (3.5)	1 (4.3)	1 (6.3)	
Unknown	10 (5.0)	1 (4.3)	1 (6.3)	
**Smoking status**				
Yes	2 (1.0)	0(0.0)	4 (25.0)	<0.0001
No	200 (99.0)	24 (100.0)	12 (75.0)	
**Alcohol usage**				
Yes	32 (15.8)	4 (16.7)	1 (6.3)	0.57
No	170 (84.2)	20 (83.3)	15 (93.7)	
**HIV infection**				
Yes	1 (0.5)	0(0.0)	0(0.0)	0.29
No	167 (82.7)	17 (73.9)	16 (100.0)	
Unknown	34 (16.8)	6 (26.1)	0(0.0)	
**Hepatitis**				
Yes	0(0.0)	1 (4.3)	0(0.0)	0.008
No	202 (100.0)	22 (95.7)	16 (100.0)	

TB, tuberculosis; pNTB, non-confirmed TB pregnant women; pATB, active TB-confirmed pregnant women; ATB, active TB-confirmed non pregnant women; BCG, Bacille de Calmette–Guerin; HIV, human immunodeficiency virus.

The clinical symptoms for each participant in pregnant women (pNTB and pATB) and ATB groups are described in **Table 2**. Cough was reported for all included pregnant women and the ATB group at inclusion (100%). One participant had HIV infection, and another had hepatitis. Both were excluded from analyses.

**Table 2:** Description of presumptive TB clinical symptoms of the study participants.

Clinical symptoms	pNTB n = 204	pATB n = 23	ATB n = 15	p value
Cough	204 (100.0)	23 (100.0)	15 (100.0)	1.00
Fever	62 (30.4)	9 (39.1)	10 (66.7)	0.01
Hemoptysis	24 (11.8)	8 (34.8)	3 (20.0)	0.009
Weight loss	95 (46.6)	19 (82.6)	15 (100.0)	<0.001
Loss of appetite	83 (40.7)	16 (69.6)	-	0.01
Night swear	69 (33.8)	14 (60.9)	13 (86.7)	0.0002
Dyspnea	96 (47.1)	14 (60.9)	-	0.70
Chest pain	73 (35.8)	9 (39.1)	11 (73.3)	0.01
Other symptoms/comorbidities	24 (11.8)	1 (4.3)	3 (20.0)	0.32

pNTB, non-confirmed TB pregnant women; pATB, active TB-confirmed pregnant women; ATB, active TB-confirmed non pregnant women.

### Concentrations of IFN-γ Production Upon *M. tuberculosis* Antigen Stimulation by Pregnancy Status

**Figure 1:**
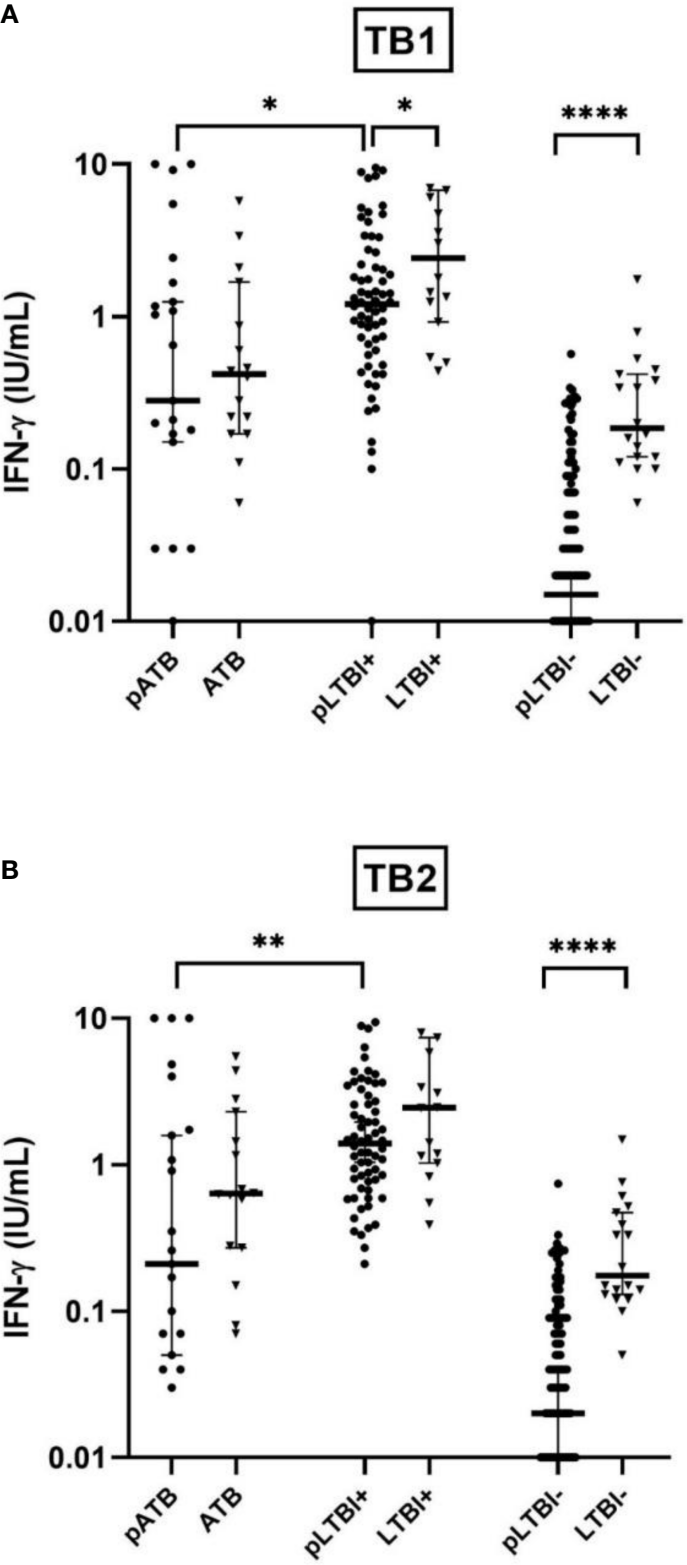
Comparison of IFN-γ production response by QuantiFERON-TB Gold-Plus Assay with stimulation with TB1 **(A)** and TB2 **(B)** antigens between pregnant women (pATB, pLTBI-, and pLTBI+) and their non-pregnant counterparts (ATB, LTBI-, and LTBI+). Symbols indicate individual IFN-γ values for pregnant participants (black circles) and non-pregnant (black triangles). The solid lines indicate median and 95% CI. The Mann–Whitney U-test (two-tailed) comparison were calculated and shown as *p < 0.05, **p < 0.01, and ****p < 0.0001. pATB, TB-confirmed pregnant women; ATB, TB-confirmed non-pregnant women; pLTBI-, pregnant women with QFT-P negative response; pLTBI+, pregnant women with QFT-P positive response; LTBI-, healthy non-pregnant control with QFT-P negative response; LTBI+, healthy non-pregnant control with QFT-P positive response.

In order to study the impact of pregnancy on the IFN-γ production upon *M. tuberculosis* antigen stimulation, the plasma IFN-γ concentrations in response to TB1 and TB2 were measured with the QFT-P assay in pregnant and non-pregnant women within the active clinical TB subgroup: pATB vs. ATB; the non-confirmed-TB pregnant women with positive QFT-P (pLTBI+) vs. the healthy non-pregnant women with positive QFT-P (LTBI+); and the non confirmed-TB pregnant women with negative QFT-P (pLTBI-) vs. healthy non-pregnant women with negative QFT-P (LTBI-) (**Figure 1**).

A globally lower median concentration of IFN-γ in response to TB1 or TB2 was observed in the pregnant women of the different clinical subgroups compared to their non-pregnant counterparts. This lower IFN-γ concentration is particularly significant (p < 0.0001) in pLTBI- compared to LTBI- with a mean IFN-γ concentration of 0.04 vs. 0.34 IU/ml with TB1 and 0.05 vs. 0.34 IU/ml, with TB2). Among the women with positive QFT-P, this difference was significant after stimulations with TB1 (respectively with a mean IFN-γ of 2.04 vs. 4.05 IU/ml, p < 0.05 for pLTBI+ vs. LTBI+), while it was non-significant after stimulations with TB2, despite a lower IFN-γ concentration in the pregnant women after stimulation of both antigens (**Figures 1A, B** **)**.

No statistically significant differences were observed when comparing the IFN-γ concentrations in the active TB group (pATB vs. ATB), despite lower concentrations of IFN-γ in the pregnant women in response to both TB1 and TB2 (**Figure 1**).

Finally, among the pregnant women, low IFN-γ magnitudes were observed in the pATB group compared to the pLTBI+ group (p < 0.05 with TB1 and p < 0.01 with TB2) (**Figure 1**).

### Comparison of *M. tuberculosis* Antigen Triggered IFN-γ Concentrations After 6 Months Follow-Up (TB Treatment and Postpartum)

**Figure 2:**
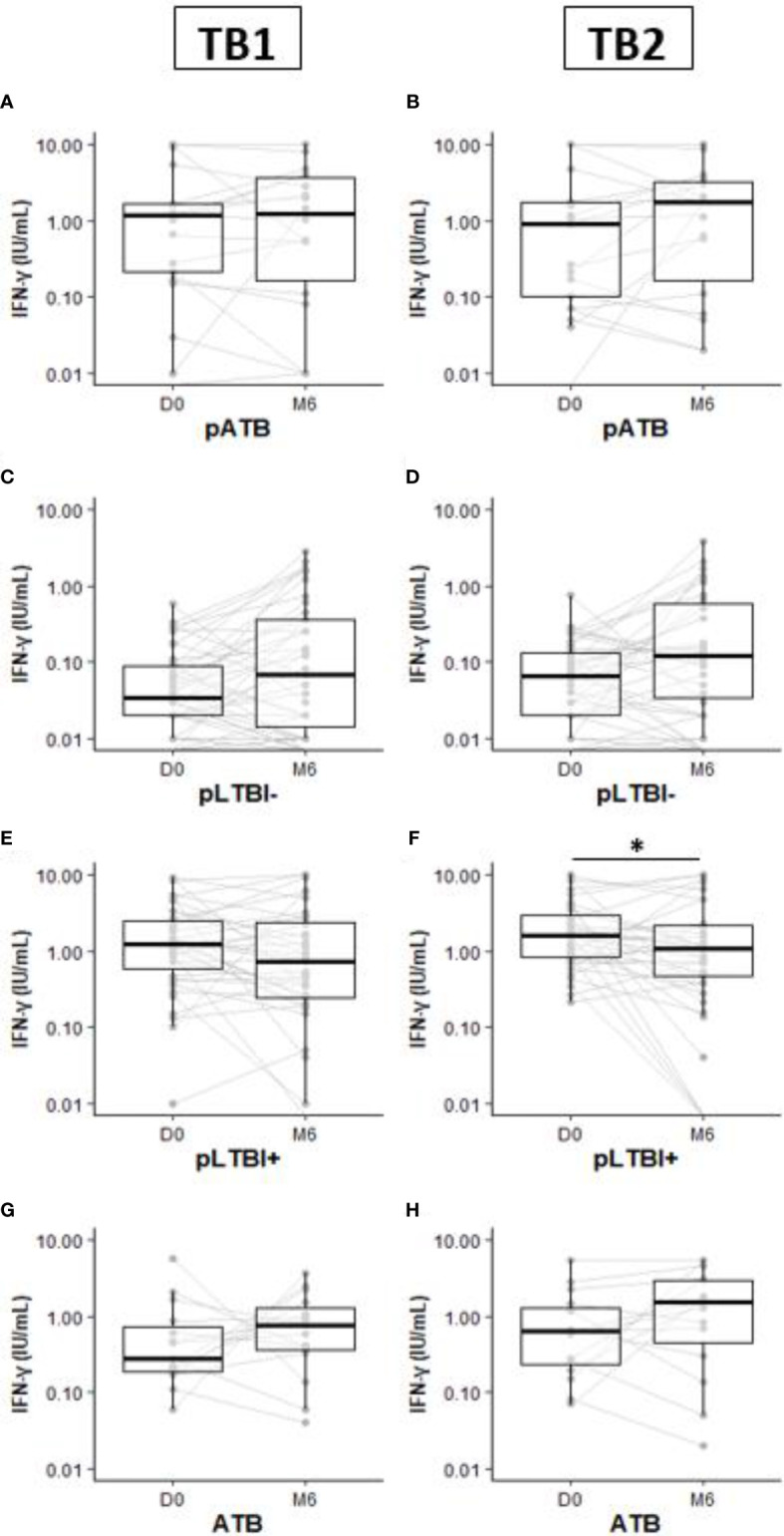
Comparison of IFN-γ production response by QuantiFERON-TB Gold-Plus Assay within pregnant and non-pregnant women subgroups pATB **(A**, **B)**, pLTBI- **(C**, **D)**, pLTBI+ **(E**, **F)**, and ATB **(G**, **H)** between inclusion (D0) and the 6-month follow-up visit (M6). Symbols indicate individual IFN-γ values. Boxes indicate median values. Wilcoxon paired test comparisons were calculated and shown as *p < 0.05. DO, inclusion; M6, six months follow up visit; pATB, TB-confirmed pregnant women; ATB, TB-confirmed non-pregnant women; pLTBI-, pregnant women with QFT-P negative response; pLTBI+, pregnant women with QFT P positive response.

All the participants confirmed of active TB successfully completed their TB treatment course, based on resolution of clinical symptoms and sputum conversion to negativity. There was no occurrence of relapse or recurrence recorded. One hundred and twenty-three (123) pregnant women (105 pNTB and 18 pATB) and 15 ATB had a 6-month follow-up visit (M6) (**Figure 2**).

At M6, although not statistically significant, increased concentrations of IFN-γ were observed in the pLTBI- group after TB1 or TB2 stimulations compared to the concentrations measured at the inclusion (D0) (**Figures 2C, D** **)**.

Among the pLTBI+, a decrease in IFN-γ concentrations in the pregnant women at M6 after stimulation with both antigens was observed compared to the concentrations measured at D0 and this difference was statistically significant with TB2 antigen stimulation (p < 0.05) (**Figure 2F**). No statistical differences in IFN-γ response magnitude were observed between measures at D0 and M6 for the pATB and ATB groups (**Figures 2A, B, G, H** **)**.

### QFT-Plus Assay Qualitative Evolution

After assigning the manufacturer threshold for qualitative positivity, a QFT-P positivity of 58.3% was observed in pATB, 34.4% in pNTB, 62.5% in ATB, and 45.7% in HC (**Table 3**).

**Table 3:** QuantiFERON-TB Gold-Plus assay results regarding the study participants clinical group.

	Total	QFT-P n (%)		
		Pos	Neg	Ind
HC	35	16 (45.7)	18 (51.4)	1 (2.8)
pNTB	209	72 (34.4)	132 (93.2)	5 (2.4)
pATB	24	14 (58.3)	10 (41.7)	0 (0.0)
ATB	16	10 (62.5)	5 (31.2)	1 (6.2)

QFT-P, QuantiFERON TB Gold Plus; pNTB, non-TB pregnant women; pATB, TB-confirmed pregnant women; ATB, non-pregnant TB-confirmed women; HC, healthy non-pregnant women; Neg, negative; Pos, positive; Ind, indeterminate.

**Table 4** shows the QFT-P assay result regarding the characteristics of pregnant women groups. In univariate analysis, it was observed that older age (p < 0.05), previous TB treatment, and previous TB contact histories (p < 0.01) were associated with positive QFT-P response. No association on QFT-P result was observed regarding pregnancy-related factors (gestational age, parity, and gravidity) and pregnancy outcomes (preterm vs. full-term birth; caesarian vs. normal vaginal birth) (data not shown).

**Table 4:** QuantiFERON TB Gold-Plus (QFT-P) assay results stratified by characteristics of the pregnant women group at the inclusion (D0).

Age	Total	QFT-P D0		*p value*
		Pos	Neg	
<20	45	13 (28.8)	32 (71.1)	
20–24	49	20 (40.8)	29 (59.1)	0.2
25–29	69	21 (30.4)	48 (68.6)	1
≥30	65	31 (47.7)	34 (52.3)	0.04
TB contact history				
Yes	52	28 (53.8)	24 (46.1)	0.0037
No	176	55 (31.2)	121 (68.8)	
TB treatment history				
Yes	20	14 (70.0)	6 (30.0)	0.0036
No	208	69 (33.1)	139 (66.8)	
Birth course				
Gave birth	125	55 (44.0)	70 (56.0)	0.48
Ongoing pregnancy	22	12 (54.5)	10 (45.5)	

TB, tuberculosis; QFT-P, QuantiFERON TB Gold Plus; Neg, negative; Pos, positive.

**Figure 3:**
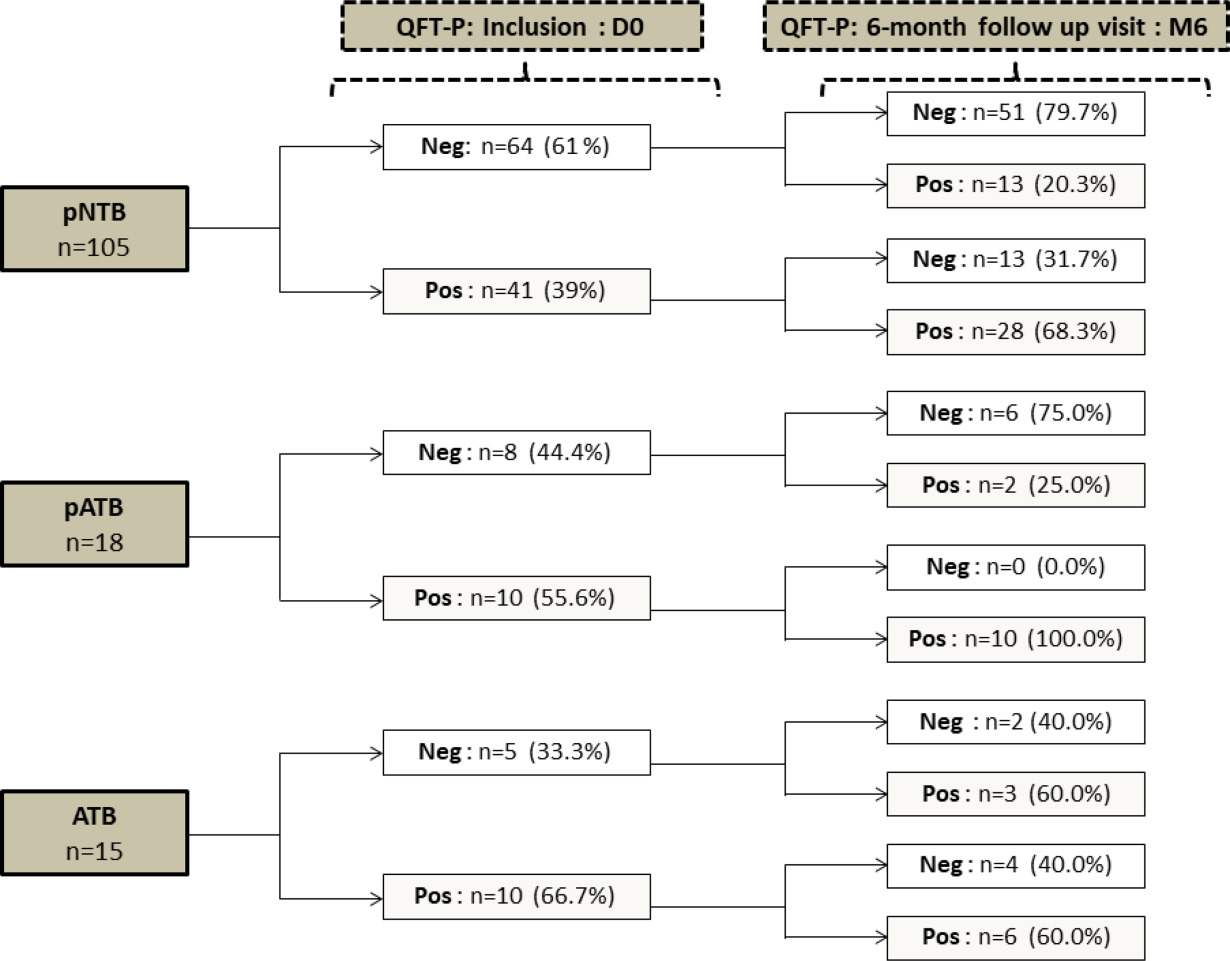
Qualitative QuantiFERON-TB Gold-Plus Assay conversion and reversion proportion within pregnant women (pNTB and pATB) and TB-confirmed non-pregnant women (ATB) groups between inclusion (D0) and the 6-month follow-up visit (M6). QFT-P, QuantiFERON-TB Gold Plus; Neg, negative; Pos, positive; DO, inclusion; M6, six months follow up visit; pNTB, non-TB pregnant women; pATB, TB-confirmed pregnant women; ATB, TB-confirmed non-pregnant women.

**Figure 3** shows the conversion/reversion rates of QFT-P assay at M6. The conversion rates were respectively 20.3% (13/64), 25% (2/8), and 60% (3/5) for the pNTB, pATB, and ATB groups, respectively. The pNTB and ATB groups had reversion rates of respectively 31.7% (13/41) and 40% (4/10), respectively. Interestingly, all the 10 pregnant women in the pATB group who had a positive QFT-P test at inclusion remained with positive QFT-P at M6 giving a reversion rate of 0% (0/10) with similar concentrations of IFN-γ production magnitude at D0 and at the end of their TB treatment (**Figure 3**).

## Discussion

This study was performed to analyze the impacts of the immune system changes during the pregnancy that might contribute to the increased risk of morbidity and mortality associated with *M. tuberculosis* exposure. We observed a decreased IFN-γ production from whole blood T cell *ex vivo* stimulation with *M. tuberculosis*-specific antigens in pregnant women independently to their clinical TB groups when compared to their non-pregnant women counterpart with similar childbearing ages. This alteration of the Th1 response in pregnant women is involved in physiological processes known to preserve the fetus (4). Here the present study showed that the concentrations of IFN-γ produced are lower in pregnant women exposed to *M. tuberculosis* (latent infection and active TB) compared to those non-pregnant with active TB (ATB) and healthy controls (HC) baseline concentrations. Moreover, in the non-pregnant asymptomatic women with negative QFT-P responses (LTBI-), this IFN-γ production is higher than in pregnant women having TB symptoms.

After being exposed to *M. tuberculosis*, the host immune system establishes the innate immune response followed by a strong implication of the cell-mediated response including the production of IFN-γ that can activate further cellular responses but can also trigger an intracellular antibacterial role (15). It has been reported that pregnancy impaired the immune response against TB by suppressing the Th1 pro-inflammatory response (16). Hormones associated with pregnancy such as progesterone were suggested to cause the placenta to produce cytokines such as IL-10 that suppress the production of Th1 cytokines, including IFN-γ (17, 18). Such changes lead then to an altered immune system status and could impair the capacity to produce TB-specific IFN-γ (19). While our study seems to confirm this global decline in the IFN-γ responses observed from other longitudinal studies in pregnant women (7, 20), the additional parallel comparison of the IFN-γ response with that of control non-pregnant women of similar age in our study showed that this IFN-γ response is strongly requested following an exposition to *M. tuberculosis* in pregnant women. Similar immune responses with a strong IFN-γ production were observed among LTBI+ pregnant women in India after the comparison with LTBI- pregnant women (10). This strong activation of IFN-γ following an exposition to *M. tuberculosis* and observations made on the antiviral immune response in pregnant woman seems to confirm the fact that pregnancy is not a period of immunosuppression notably for Th1 but a temporary attenuation in immune responses characterized by a strengthening of innate immune barriers and a concomitant reduction in pro-inflammatory immunity that is resumed when exposed to infections (21, 22).

After delivery, the IFN-γ magnitude seems to return to its baseline concentration except for the former active TB group that was successfully treated in which the *M. tuberculosis*-specific IFN-γ response remains high and positive regarding the QFT-P response. All the previous active TB pregnant women (pATB) remained indeed positive after 6 months of follow-up with the QFT-P assay. This phenomenon could be explained by the postpartum immune reconstitution syndrome (23). Physiologic or enhanced proinflammatory response (especially Th1) during the postpartum period and reversal in the cytokine pattern 3–6 weeks after delivery were also previously observed (24). However, as the study was conducted in a high TB incidence area, a reinfection or a continuous exposition to TB has to be considered especially in those that had the TB symptoms.

Finding TB-related symptoms in pregnant women that are immunoreactive to *M. tuberculosis* antigen but without any bacteriological confirmation (pLTBI+) may indicate an immune control of the subclinical form of the disease that is limiting the active replication of the bacteria (25). On the other hand, the observed decline in IFN-γ after 6 months of follow-up in those non-confirmed TB pregnant women with positive QFT-P may explain the risk of postpartum TB already observed in populations from high-incidence areas as is the case in Madagascar for this study (8, 26, 27). However, according to our observations, despite a global decrease in the IFN-γ response during pregnancy, a sufficiently protective IFN-γ response seems to be protective and allows to contain an infection because even after a 6-month follow-up, no women with positive QFT-P did develop any active TB disease form.

Lastly, due to the pregnancy-associated immunologic and physiologic changes that had been reported to have influence on the performance of IGRA (28, 29), the choice of a threshold concentration for definition of a positive reaction for QFT-P has been debated, especially since results close to the recommended cutoff concentration (0.35 IU/ml) can be subject to variability. In the current study, we assumed the use of the recommended cutoff concentration of 0.35 IU/ml rather than a lower threshold concentration as suggested by other researchers (30) to allow the assessment of longitudinal variations and comparison of the IFN-γ concentrations with non-pregnant control that would be difficult with specific cutoff concentration per clinical group.

Our study has several limitations. In the present study, only four pregnant women in the first trimester of pregnancy were enrolled and the majority of the participants in study were recruited at their second and third trimesters of pregnancies. This did not allow further robust statistical comparisons of IFN-γ concentrations regarding gestational age. The gradually occurring changes in cytokine production including IFN-γ responses over the course of the pregnancy have been reported to be more pronounced in the second and third trimesters (8, 21). Second, we have not investigated other TB-specific cytokines. There are changes among other cytokines that are specific to pregnancy (10). The next steps for this study would be to better understand the impact of these variations by assessing other cytokines and biomarkers of LTBI and/or progression to active TB. Third, due to the sample size and the limited data collected from the participants, we did not investigate the association between the increase in IFN-γ concentration and the pregnancy outcomes (delivery mode, birth term, infant outcomes) until the 6-month follow-up visits. However, in this study we found that the proportion of caesarean delivery was higher in those pregnant women with an elevated IFN-γ response independently of the TB clinical issue (data not shown).

To conclude, this study gives an overview of the immune response dynamics during pregnancy and postpartum in *M. tuberculosis*-infected women. Our findings suggested that despite the decline in IFN-γ response observed during pregnancy, a high concentration of *M. tuberculosis*-induced IFN-γ production was observed in pregnant women with a controlled infection and a lower concentration of this cytokine is observed in those with active TB that however triggers persistent positive QFT-P after the TB treatment. Early detection of *M. tuberculosis* infection followed by preventive treatment administration was suggested to be critical to the global TB control and eradication efforts. More attention should be made with pregnant women as they had a specific immune status and could be vulnerable to *M. tuberculosis* infection and reactivation. These findings suggest a need for implementation of improved strategies to scale up *M. tuberculosis* infection screening among high-risk pregnant women to advance in TB elimination and/or impact the TB-related morbidity and mortality especially in those high-risk pregnant women and their infants in high burden settings.

## Data Availability Statement

The raw data supporting the conclusions of this article will be made available by the authors on request to the corresponding author.

## Ethics Statement

The studies involving human participants were reviewed and approved by Comité d’éthique pour la recherche Biomédicale of the Ministry of Public Health of Madagascar. Written informed consent to participate in this study was provided by the participants’ legal guardian/next of kin.

## Author Contributions

NR and PR conceived the idea of this article and provided the framework. All authors collected and analyzed the relevant information. PR undertook the statistical analysis. PR wrote the first draft of the manuscript. The initial draft was revised by all authors. All authors read and approved the final manuscript. All authors contributed to the article and approved the submitted version.

## Funding

This study has received funding from the Grand Challenges Africa [GC Africa Innovation Seed Grant number GCA/ISG/rnd1/115].

## Conflict of Interest

The authors declare that the research was conducted in the absence of any commercial or financial relationships that could be construed as a potential conflict of interest.

## Publisher’s Note

All claims expressed in this article are solely those of the authors and do not necessarily represent those of their affiliated organizations, or those of the publisher, the editors and the reviewers. Any product that may be evaluated in this article, or claim that may be made by its manufacturer, is not guaranteed or endorsed by the publisher.
